# Effect of cryotherapy, laser therapy and intraligamentary dexamethasone injection on post-treatment pain after single visit RCT–A double-blinded, randomized placebo-controlled trial

**DOI:** 10.1016/j.jobcr.2025.01.005

**Published:** 2025-02-09

**Authors:** Sana Fatima, Sonali Taneja, Vivek Aggarwal, Akriti Dheer, Punya Taneja

**Affiliations:** a Department of Conservative Dentistry & Endodontics, I.T.S Centre for Dental Studies & Research, Ghaziabad, India; b Faculty of Dentistry, Jamia Millia Islamia, India; c Sardar Patel Institute of Dental & Medical Sciences, Lucknow, India

**Keywords:** Cryotherapy, Low-level laser therapy, Post endodontic pain, Single-visit root canal treatment, Symptomatic apical periodontitis

## Abstract

**Aim:**

This study designed to evaluate the effect of adjuvant therapies on the intensity of post-treatment pain after single-visit RCT in molars with symptomatic apical periodontitis.

**Method:**

ology: This trial was reported according PRIRATE 2020 guidelines and registered with CTRI/2019/03/018,153.100 patients diagnosed with symptomatic apical periodontitis with a preoperative pain allocated to 4 groups,Group-1: placebo,Group-2:Intraligamentary injection of dexamethasone, Group-3:cryotherapy, Group-4: laser therapy. Patients were instructed to record postoperative pain intensity and intake of analgesics after 6,12,24,48 & 72 h. Intergroup pain intensity was tested by using the Kruskal-Wallis test. The need for painkillers between groups was analyzed using Chi-square test. P value of <0.05 was considered statistically significant.

R**esults**: Patients in the placebo group exhibited higher intensity. Group 3 showed lesser pain at all-time intervals.

**Conclusion:**

All the adjuvant pain management therapies reduced the intensity of postoperative pain and the frequency of intake of analgesics.

## Graphical abstract

**Figure:**
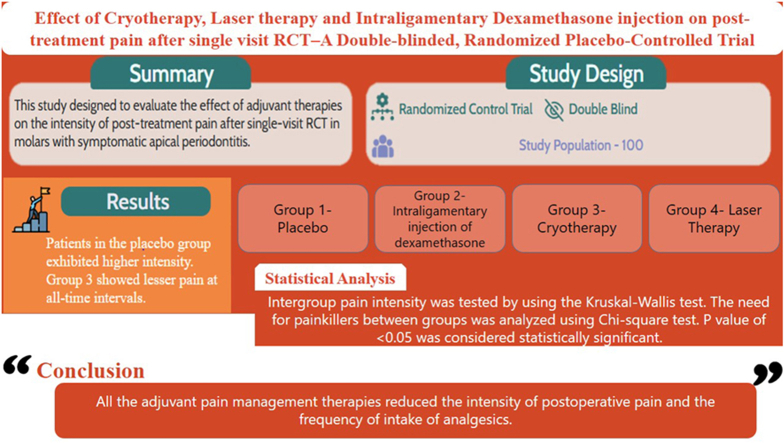

## 1 Introduction

Pain following a root canal procedure (RCT) is an undesirable consequences, both for the patient and the clinician. The neuronal response to the production of chemical mediators of acute inflammation, including cytokines, prostaglandins, leukotrienes, bradykinin, and serotonin, which sensitise and activate the nociceptors, is what causes the patient to experience pain..^1^ The incidence of pain after an endodontic therapy has been reported to be dependent on multiple variables.^2^ Hargreaves and Hutter (2002) elaborated that post-endodontic pain can be anticipated in teeth presenting with symptomatic apical periodontitis, pulp necrosis, and preoperative pain 3. Arias *et-al* (2013) studied the correlation between post-endodontic pain, tooth diagnostic factors, and apical patency and deduced that postoperative pain increased in necrotic pulps with apical periodontitis as compared to vital pulps because of inflammation of the periodontium^4^

The most routinely employed pain alleviating measure in the aftermath of a root canal treatment is the prescription of systemic analgesics, specifically NSAIDS.^5^ Using local pharmaceutical therapy to reduce post-operative endodontic discomfort and give the inflamed tooth the necessary anti-inflammatory impact is another viable method..^6^ Evidence from available dental literature advocate that drugs like corticosteroids, via oral, intramuscular or intraligamentry routed has shown reduction in the frequency of postoperative pain for up to 24 h after the RCT.^7^ Deposition of dexamethasone or prednisolone locally near th site of inflammation, via the intraligamentary route has proven to be effective analgesic due to its potent anti-inflammatory action because of inhibition of cytokine and prostaglandin production.^8^ An emerging non-pharmacological pain management therapy in endodontics is Cryotherapy.^9^ It has previously been used for the management of pain in sports injuries and surgical procedures. In order to manage postoperative pain, cold therapy is used during intraoral surgical procedures.

The intracanal cryotherapy procedure was described for the first time by Vera et al. (2015) and further proven that it was effective in a significant reduction of post-endodontic pain in teeth with symptomatic apical periodontitis.^10^^,^^11^

Lasers are another advancement which has gained hold in pain management therapy. The photo bio modulation by Low-level laser therapy (LLLT) reduces post-operative pain and swelling due to its anti-inflammatory and neuro-regenerative effect^12^ In endodontics, it has proven to be beneficial in reducing post endodontic pain in molars with symptomatic apical periodontitis.^13^

Although various clinical trials have been conducted comparing the efficacy of analgesics in the reduction of post-endodontic pain 14, no trial has yet been done comparing the efficacy of the above described adjuvant therapies. The research question was framed using the following framework- Population: patients with symptomatic apical periodontitis; Intervention and comparative agent: cryotherapy, intraligamentary injection of dexamethasone, LLLT, and control group; Outcome analysis: post-operative pain levels.

The purpose of this randomised controlled experiment is to compare and assess the consequences of three adjuvant therapy modalities on the degree of discomfort following a single-visit root canal procedure: cryotherapy, low-level laser therapy, and intraligamentary injection of dexamethasone. As per the null hypothesis patient with symptomatic apical periodontitis getting cryotherapy, intraligamentary injection of dexamethasone, LLLT, and a control group would have the same postoperative pain levels.

## 2 Methodology

The present study was conducted after taking the necessary ethical clearance from the institutional ethical committee (ITSCDSR/L/2019/006) in the Dept. of Conservative Dentistry and Endodontics at I.T.S. Centre for Dental Studies and Research, Muradnagar, Ghaziabad. The study was designed as a randomized, placebo-controlled, single-center, double-blinded trial.

The trial was also registered with the Clinical Trials Registry- India, (CTRI/2019/03/018,153). Informed consent was taken from every patient before enrolling them in the study. The Preferred Reporting Items for Randomized Trials in Endodontics (PRIRATE) 2020 standards were followed in the formulation of the current trial..^15^ The trial was conducted from March 22, 2019 to November 28, 2019.

A pilot study of 10 patients revealed the expected standard deviation of pain score of Control (Group I) and Intraligamentary Injection (Group II) as 1.308, 1.251 respectively, mean scores 4.38 and 3.36 respectively at 6 h and therefore a mean difference of 1.02. Using the given formula software Open Epi, Version 3^16^**,** a total sample size of 100 was calculated with 25 patients in each group. The sample size was determined using a 5 % alpha error and 80 % statistical power.

n=(12+22)(Z1−a2+Z1−β)22

The notation for the formulae is:

n = sample size of Groups

s1 = standard deviation for Group 1 = 1.308

s2 = standard deviation for Group 2 = 1.251.

D = difference between group means = 1.02.

Z1-α/2 = two-sided Z value (eg. Z = 1.96 for 95 % confidence interval).

Z1-β = power = 80 %

## 3 Selection of patients

Postgraduate dental student examined a total of 144 individuals., unrelated to the trial. Hundred patients who were diagnosed with necrotic pulps and symptomatic apical periodontitis and agreed to participate in the study were selected. The Diagnosis was made by pulp sensibility test and intra oral peri apical radiographs. A combination of cold test (Roeko Endofrost, Coltene), Germany and electric pulp test (API, India) were used to make the pulpal diagnosis. Diagnosis of symptomatic apical periodontitis was made by a positive response to tenderness on palpation, percussion and mastication.

The Patients were instructed to fill Visual Analogue scale (VAS) which included a 10 cm straight horizontal line with numbers at each centimeter; 0 No pain, 1–3 mild pain, 4–6 moderate pain, 7–9 severe pain, 10- worst pain possible. Teeth with severe spontaneous pain score of ≥7 (VAS) were selected for the trial.

### 3.1 Inclusion criteria

Systemically healthy (ASI I) patients, aged 18–65years, diagnosed with symptomatic apical periodontitis in a mandibular molar needing primary endodontic procedure with a preoperative VAS score of ≥7.

### 3.2 Exclusion criteria

Exclusions from the trial included patients with a history of allergy to any of the experimental drugs or local anaesthetic solutions, patients who were medically compromised, and patients who had taken steroids, antibiotics, or analgesics within the previous 24 h.

## 4 Randomization and blinding

An uninvolved person conducted the allocation concealment and random sequence creation. Using the randomization software (www.randomizer.org), four sets of twenty-five patients were allocated at random to a permuted block. Sealed opaque envelopes were numbered with the codes generated by the software and the patients were randomly assigned to any of the 4 groups.

Group 1: Control group (Placebo).

Group 2: Intraligamentary injection with dexamethasone group.

Group 3: Cryotherapy group.

Group 4: Low-Level Laser Therapy (LLLT) group.

The coded envelopes were decoded only at the end of the clininal trial.

Randomization assured the external validity of the study, allowing for the generalizability of result. The internal validity was assured by using a control (placebo) group.

By keeping both the patient and the outcome evaluator being unaware about the intervention employed, double blinding was accomplished**.**

## 5 Intervention and clinical procedure

All treatments were performed by a single operator. The patients were blinded to the therapy being used. LA injection of 1.8 mL of 2 % lidocaine (Xicaine, ICPA India) containing 1:80,000 epinephrine was given following the recording of the preoperative pain levels. To determine of the depth of anesthesia two test were conducted via a cold test (Roeko Endofrost, Coltene, Germany) and an electric pulp tester (API, India) at 15-min intervals.

After the anesthesia, for the patients in Group 2, 0.2 mL of dexamethasone was injected into the periodontal ligament (PDL) at the tooth's mesiobuccal and distobuccal corners, with the needle inserted at a 45-degree angle into the PDL. In the other groups, the operator pretended to perform a PDL injection. The Injection filled with saline only touched the tissue without any penetration and the saline was emptied into a cotton roll placed in the buccal vestibule adjacent to the tooth.

Dental dam was applied and occlusal reduction was followed by preparation of the access cavity in all the patients using sterile burs. Working length determination was done by using an electronic apex locator (Root ZX mini, J Morita Corp) and further established with an intraoral periapical radiograph. Protaper Gold rotary files (Dentsply-Tulsa, Switzerland) were used to widen the canals to a minimum of size 25(0.08). Between instruments, 2 ml of 3 % NaOCl was used for irrigation, (Parcan; Septodont, India) with a 31-gauge Navitip needle (Ultradent Products Inc, USA). After the root canal preparation was completed, the irrigant was activated using an Irrisafe ultrasonic 20.00 tip (Satelec, Merignac, France) with an ultrasonic unit (Satelac- Acteon, USA) at half its power. The activation was done three times, each lasting 20 s, with the tip positioned 3 mm away from the WL. As a last irrigant, 17 % EDTA was added to the canal up to 1 mm below the working length, and it was left there for a minute. In Group 3 (Cryotherapy group), cold normal saline at a temperature of 2.5 °C was used as an irrigant. The saline was refrigerated until use. The temperature was checked by a thermometer and the irrigation needles interimly stored in an ice box between use the cold saline was irrigated 2 mm short of the working length for 5 min and 20 ml was placed in the root canals using a 31 G Navi-Tip needle. In the other groups normal saline at room temperature was used.

The root canals were then dried with sterile paper points and the canals were obturated with matched single cones gutta-percha (Dentsply Maillefer, Switzerland) and AH Plus (Dentsply Maillefer, Switzerland) sealer.

Restoration of the cavity was done with a resin composite (Filtek TM Z350 universal, USA) and the occlusion and proximal integrity were checked to prevent future pain.

Patients of Group 4(LLLT) received laser irradiation using a diode laser (Biolase epic X, USA).

Diode lasers with wavelengths of 940 nm were used to irradiate the LLLT patient. A bleaching application tip that was positioned 10 mm away from the tissue and a 200-mm optical fibre were used in the application. The mesial and distal apical regions of the molar were given radiation at 0.5W for 30 s each. In the other groups, the laser tip was placed but not activated.

In group 1: Control (Placebo) - An injection with saline was emptied into a cotton roll placed in the buccal vestibule adjacent to the tooth after administering local anesthesia. Final irrigation was performed with normal saline at room temperature for 5 min and the LLLT laser tip was placed but not activated.

A prescription of Ibuprofen 400 mg (Brufen; Abbott, India) was provided to the patients to be taken only in cases of severe pain and they were instructed to record the intake frequency.

## 6 Monitoring of patients and outcome assessment

Patients received a VAS scale book with many VAS scales corresponding to various time points. The investigator also had a similar scale with them, and they compared the VAS pain signals the patient noted with the appropriate scores ranging from 0 to 10. The patients were called by a blinded investigator at all the scheduled times, and they were informed beforehand that they needed to fill out the pain questionnaire after 6, 12, 24, 48, and 72 h.

Additionally, the patients were asked to document how often they took analgesics to manage their pain following procedure.

## 7 Statistical analysis

The SPSS statistical program (IBM Corp., version 24.0, Armonk, NY, USA) was used to analyse the data. Non-parametric tests were used to determine the significance after the Shapiro-Wilk test revealed that the data did not follow a normal distribution. The Kruskal-Wallis test was used to measure the pain intensity between the four groups (intergroup comparison), and the Dunn Man Whitney *U* test was used after that. Friedman's test was used to measure the intensity of the pain within the similar group at the various time intervals (intragroup comparison).

The variables such as membership of group, age, gender, number of teeth, preoperative pain levels, and VAS pain ratings on percussion that were most closely associated with postoperative pain were assessed using linear regression analysis as dependent variables are quantitative. Using the Pearson's Chi-square test, analgesic intake was compared between groups.

A 95 % confidence interval and a p-value less than 0.05 were considered significant (p < 0.05).

## 8 Results

**Fig. 1:**
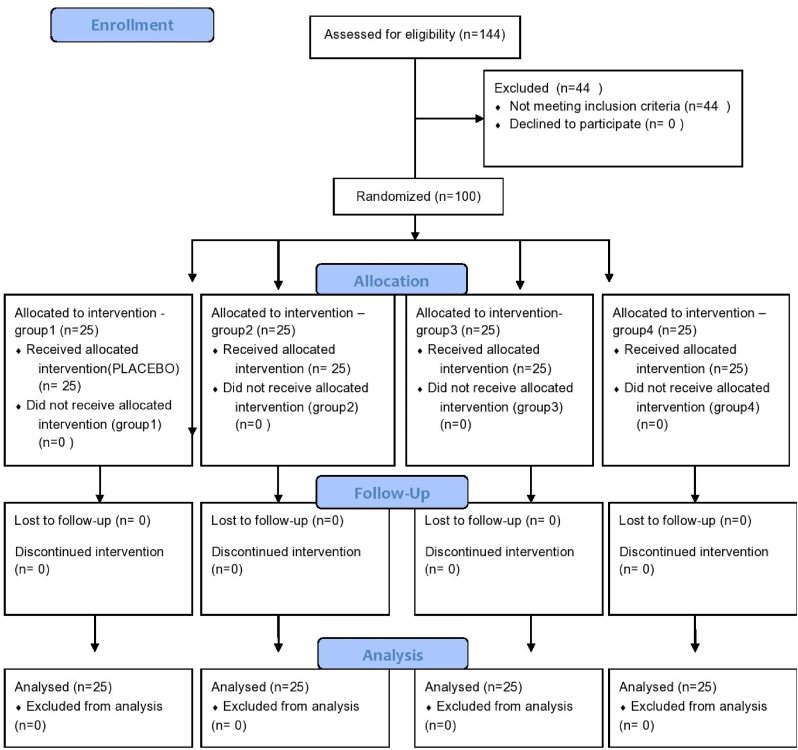
Flow diagram (CONSORT 2010).

This trial was conducted from March 22, 2019 to November 28, 2019. The PRIRATE 2020 flowchart denotes the participant flow during each of the trial phases (Fig. 1). Single visit RCT was done in one hundred mandibular molars. Statistical analysis revealed, no significant difference in baseline data of the hundred patients in terms of preoperative pain levels (p = 0.498), gender (p = 0.654), age (p = 0.805), and tooth number (p = 0.989) (Table 1) (see Graph 1).

**Graph- 1:**
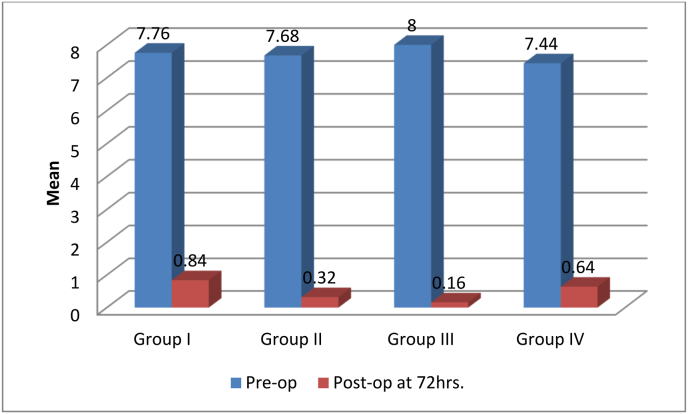
Comparision of means Pre-op and Post- Op (72 h) Pain intensity among four groups

**Table 1:** Baseline Demographics of distribution of patients among the groups.

Variables	1: Control group (Placebo)	2: Intraligamentary injection with dexamethasone group	3: Cryotherapy group	4: Low-Level Laser Therapy (LLLT) group	p-value
**Age (Mean, SD)**	46.12 (16.627)	42.76 (13.887)	42.76 (16.839)	43.04 (13.670)	0.805
**Gender n (%)**					
**Male**	12 (48.0)	10 (40.0)	9 (36.0)	13 52.0 %	0.654
**Female**	13 (52.0)	15 (60.0)	16 (64.0)	12 (48.0)	
**Tooth Type n (%)**					
**First Molar**	15 (60.0)	15 (60.0)	14 (56.0)	15 (60.0)	0.989
**Second Molar**	10 (40.0)	10 (40.0)	11 (44.0)	10 (40.0)	
**Pre-op Vas Score (Median, IQR)**	8.00 (2)	7.00 (3)	8.00 (2)	7.00 (3)	0.498

# SD– Standard deviation,∗p < 0.05 significant, ∗∗p < 0.01 Highly Significant, VAS–Visual Analog Scale, Pre op–Preoperative, IQR–Interquartile range.

Overall pain intensity decreased post-operatively (over-all mean of 3.20 at 6 h and 2.16 at 24 h). However, the intensity of post-operative pain in the placebo group was higher than the intervention groups at all-time intervals (Table 2). Intensity of Pain was significantly lower in the cryotherapy group compared to the other groups at 6, 12, 24 and 48 h post-operatively. The pain levels dropped to below clinical relevance after 12 h in the cryotherapy group and in 48 h in placebo group. Groups 2 and 4 showed higher pain levels than the cryotherapy group and lower than the placebo group at all-time intervals. At 6–12 h postoperatively, the LLLT (group4) demonstrated significantly lower pain levels than the Intraligamentary injection with dexamethasone group (group2) and the difference became insignificant at 24 h. The mean ± SD of pain at 72 h for Group I was 0.84 ± 0.746 (0.53–1.15), Group II 0.32 ± 0.476(0.12–0.52), Group III 0.16 ± 0.374 (0.01–0.31) and for group IV 0.64 ± 0.490(0.44–0.84) and by Kruskal-Wallis test the difference of pain intensity between four groups were highly significant p < 0.01. Hence, the pain intensity at 72hrs in Group III is significantly lower as compared to other groups. (Table 2)(Graph 1).

**Table 2:** The percentage of each group's postoperative pain at 6, 12, 24, 48 and 72 h (Mean, median, IQR).

The percentage of each group's postoperative pain at 6, 12, 24, 48 and 72 h (Mean, median, IQR).		1.Control group (Placebo)	2. Intraligamentary injection with dexamethasone group	3. Cryotherapy Group	4. Low level Laser Therapy (LLLT) Group
**6 h**	**Mean (SD)** **(95%CI)**	4.72^aA^ (1.308) (4.18–5.26)	3.36^aB^ (0.810) (3.03–3.69)	1.92^aC^ (0.702) (1.63–2.21)	2.80^aD^ (1.323) (2.25–3.35 --)
	**Median**	5.00	3.00	2.00	2.00
	**IQR**	**2**	**1**	**1**	**2**
**12h**	**Mean (SD)** **(95%CI)**	3.12^bA^ (0.726) (2.82–3.42)	2.44^bB^ (0.583) (2.20–2.68)	1.08^bC^ (0.759) (0.77–1.39)	2.00^bD^ (0.913) (1.62–2.38)
	**Median**	3.00	2.00	1.00	2.00
	**IQR**	**0**	**1**	**2**	**1**
**24 h**	**Mean (SD)** **(95%CI)**	1.88^cA^ (0.927) (1.50–2.26)	1.16^cB^ (0.688) (0.88–1.44)	0.28 ^cC^ (0.458) (0.09–0.47)	1.44)^cB^ (0.507) (1.23–1.65)
	**Median**	2.00	1.00	0.00	1.00
	**IQR**	**1**	**1**	**1**	**1**
**48 h**	**Mean (SD)** **(95%CI)**	1.00^dA^ (0.866) (0.64–1.36)	0.52^dB^ (0.586) (0.28–0.76)	0.16^cC^ (0.374) (0.01–0.31)	1.12^Da^ (0.600) (0.87–1.37)
	**Median**	1.00	0.00	0.00	1.00
	**IQR**	**2**	**1**	**0**	**0**
**72 h**	**Mean (SD)** **(95%CI)**	0.84^dA^ (0.746) (0.53–1.15)	0.32^eB^ (0.476) (0.12–0.52)	0.16^cB^ (0.374) (0.01–0.31)	0.64^eA^ (0.490) (0.44–0.84)
	**Median**	1	0	0	1
	**IQR**	**1**	**1**	**0**	**1**

#SD – Standard Deviation.

Same lower-case letters indicate no significant difference between the intergroup analysis at a time interval. Same upper-case letter indicates no significant difference between the various time intervals in intragroup comparison.

Linear Regression analysis included factors which could have an effect on the postoperative pain and it showed group variation to be the most significant factor. Sex, age, tooth number (first or second mandibular molar), and medicine intake had no significant effect on postoperative pain (p > 0.05) (Table 3). By Multinomial logistic regression, postoperative pain was the dependent variable and group was independent variable. By Ward statistics, the regression coefficient of group 2, group 3 were significant. The patient group 1 as compared to group 4 had 1.031 times more chances lies in patient with absent pain than patient with present pain. Group 3 has 11.28 chances of absence of pain therefore it showed best result (Table 3).

**Table 3:** Linear Regression Analysis of various factors on post-operative pain.

	Unstandardized Coefficients		Sig.
	B	Std. Error	
(Constant)	0.176	0.294	0.552
Group	0.573	0.045	0.001∗∗
Gender	0.176	0.088	0.050
Age	−0.002	0.003	0.449
Tooth number	−0.072	0.083	0.388
Pre-operative	−0.010	0.038	0.798
6 h	0.080	0.056	0.156
12 h	−0.112	0.075	0.143
24 h	−0.036	0.080	0.655
48 h	0.229	0.080	0.103
medicines taken	0.032	0.059	0.593

#B- Regression coefficient, ∗p < 0.05 significant, ∗∗p < 0.01 Highly Significant.

The highest amount of analgesic consumption at 6 h postoperatively was appreciated in the Control Group (76 %) and the lowermost amount was seen in the Cryotherapy Group (40 %). After 24 h, only Group 1 (control) showed analgesic consumption of 4 %. After 48 h, there was no analgesic consumption in any group (Table 4).

**Table 4:** Analgesic consumption for each group at different time intervals. Inter-group comparison of analgesic consumption between Group 1 (Control/Placebo), Group 2 (Intraligamentary injection of dexamethasone), Group 3 (Cryotherapy) and Group 4 (Low -level Laser Therapy) after 6 h, 12 h, 24 h, 48 h and 72 h.

GROUPS	AFTER 6 HRS	AFTER 12 HRS	AFTER 24 HRS	AFTER 48 HRS	AFTER 72 HRS
	YES	YES	YES	YES	YES
1 (Control/Placebo)	19 (76 %)	12 (48 %)	1(4 %)	0 (0 %)	0 (0 %)
2(Intraligamentary injection of dexamethasone)	17 (68 %)	7 (28 %)	0(0 %)	0 (0 %)	0 (0 %)
3 (Cryotherapy)	10 (40 %)	2 (8 %)	0(0 %)	0 (0 %)	0 (0 %)
4(Low-level laser therapy).	16 (64 %)	6(24 %)	0(0 %)	0 (0 %)	0 (0 %)
Pearson chi square (p value)	7.640 (0.054)	10.299 (0.016∗)	NA	NA	NA

∗p < 0.05 significant, ∗∗p < 0.01 Highly Significant, NA-not applicable as p value could not be calculated.

## 9 Discussion

Randomized double-blind placebo-controlled studies are considered the gold standard in medical and dental research as they minimize bias and offer robust evidenc.^17^ This prospective, single-center, double-blinded, randomized clinical trial was conducted to evaluate the effectiveness of adjuvant pain management therapies in reducing postoperative pain following root canal treatment (RCT) in patients with symptomatic apical periodontitis.

Generally, post-treatment pain after RCT remains mild and typically resolves within three days, although some patients experience moderate to severe pain, especially in cases of symptomatic apical periodontitis.^18^ This pain is multifactorial, often resulting from inflammatory responses to debris or filling materials that can trigger inflammatory mediators such as prostaglandins, which activate nociceptors and cause pain.^19^

In this study, postoperative pain in the placebo group (Group 1) was significantly higher and more prolonged than in the experimental groups. Specifically, cryotherapy (Group 3) showed superior outcomes, significantly reducing pain (p < 0.05) and the need for analgesics compared to the control and other experimental groups. This aligns with existing literature, where cryotherapy has been shown to reduce postoperative pain and edema by inducing vasoconstriction and decreasing local metabolism.^20^, ^21^, ^22^, ^23^ Studies by Arslan (2018), Gundogdu (2018), and Vera et al. (2018) have similarly reported lower pain scores and reduced analgesic consumption following cryotherapy after endodontic therapy.^11^^,^^24^^,^^25^

The LLLT group also demonstrated significantly lower pain levels, likely due to the laser's effect on cellular metabolism and microcirculation, enhancing healing while reducing inflammation. This finding is consistent with prior research, where LLLT has been reported to modulate inflammatory processes and reduce acute pain following RCT.^26^ Studies by Yıldız and Arslan (2018) and Bjordal et al. have shown that LLLT can reduce pain and inflammation during endodontic treatments.^27^ However, a study by Asnaashari et al. (2017) found no significant difference between LLLT and placebo groups, which suggests that the effect of LLLT may vary depending on treatment protocols or patient characteristics.

In contrast, intraligamentary dexamethasone injection (Group 2) was less effective in reducing postoperative pain compared to cryotherapy and LLLT, but it still provided some benefit over the placebo. This finding is consistent with previous studies demonstrating the effectiveness of dexamethasone in reducing postoperative pain by alleviating acute inflammation.^28^ Research by Mehrvarzfar et al. (2016) and Shantiaee et al. has shown that dexamethasone injections significantly reduce pain following RCT.^28^ Although less effective than cryotherapy and LLLT in this study, it remains a valuable option, especially in patients with more severe pain or inflammation.

The age and sex of the patients did not significantly influence postoperative pain in this study, supporting findings from Polycarpou et al. (2005) and Ng et al. (2004), who also found no strong correlation between sex and pain perception.^29^, ^30^, ^31^ However, some studies have indicated that women may report more postoperative pain than men, suggesting that gender might influence pain perception in certain populations.

The clinical implications of this study are significant, as it demonstrates that cryotherapy, LLLT, and dexamethasone injection can provide effective alternatives or adjuncts to traditional analgesic therapy in managing postoperative pain after RCT. Cryotherapy and LLLT, in particular, offer non-invasive options with minimal side effects, providing an opportunity to reduce reliance on pharmacological treatments and improve patient outcomes. The findings support incorporating these therapies into clinical practice for better pain management and enhanced patient comfort. While dexamethasone showed some efficacy, its role is more limited compared to cryotherapy and LLLT but remains a valuable option for patients who may need additional anti-inflammatory effects in the immediate postoperative period.

Several limitations must be acknowledged. First, the strict inclusion criteria limited the study to patients with symptomatic apical periodontitis, making the results less generalizable to other endodontic conditions, such as asymptomatic or necrotic pulp. Second, the use of the Visual Analog Scale (VAS) for pain assessment is subjective and can vary depending on individual pain thresholds and psychological factors. Third, potential confounding factors such as anxiety, previous pain experiences, or psychological factors were not considered, which could influence pain perception. Lastly, the small sample size and single-center design limit the external validity of the findings. Future studies with larger, multi-center samples and longer follow-up periods would help validate these results and better understand the long-term efficacy of these therapies.

## 10 Conclusion

Adjunctive therapies like Cryotherapy, LLLT, and Intraligamentary injection of dexamethasone have shown to be beneficial in reducing postoperative endodontic pain in mandibular molars with symptomatic apical periodontitis. Among these three, cryotherapy showed significantly lower pain levels at all-time intervals.

## Consent to participate

All participants enrolled signed the informed consent agreeing with their participation in the study and data usage for the publication.

## Ethical approval

The article follow the protocol established by the CONSORT statement.

## Authors contribution

Fatima S. contributed to data acquisition, Literature search & Experimental studies analysis, and interpretation, drafted and critically revised the manuscript; Taneja S. contributed to Conceptualization, designing of the study, data acquisition, analysis, data interpretation, and drafted and critically revised the manuscript; Aggarwal V. contributed to conception, design, data acquisition, and critically revised the manuscript; Dheer A. contributed to interpretation, and data acquisition and critically revised the manuscript; Taneja P. contributed to interpretation and critically revised the manuscript; All authors gave final approval and agree to be accountable for all aspects of the work.

## Clinical relevance

Cryotherapy Efficiently lowers pain levels at all-time intervals during Single Visit Root Canal Treatment. This is the one of its only study comparing Pharmacological method to Non- Pharmacological technique for relieving pain.

## Trial registration

The trial was also registered with the Clinical Trials Registry- India, (CTRI/2019/03/018,153). Informed consent was taken from every patient before enrolling them in the study.

## Funding

No funding was obtained for this study.

## Declaration of competing interest

The authors declare that they have no known competing financial interests or personal relationships that could have appeared to influence the work reported in this paper.

## References

[1] Yam M.F., Loh Y.C., Tan C.S., Khadijah Adam S., Abdul Manan N., Basir R. (2018 Jul 24). General pathways of pain sensation and the major neurotransmitters involved in pain regulation. Int J Mol Sci.

[2] Sadaf D., Ahmad M.Z. (2014 Dec). Factors associated with postoperative pain in endodontic therapy. Int J Biomed Sci.

[3] Hargreaves K.M., Hutter J.W., Cohen S., Burns R. (2002). Pathways of the Pulp.

[4] Arias A., de la Macorra J.C., Hidalgo J.J., Azabal M. (2013 Aug). Predictive models of pain following root canal treatment: a prospective clinical study. Int Endod J.

[5] Smith E.A., Marshall J.G., Selph S.S., Barker D.R., Sedgley C.M. (2017 Jan). Nonsteroidal anti-inflammatory drugs for managing postoperative endodontic pain in patients who present with preoperative pain: a systematic review and meta-analysis. J Endod.

[6] Pozzi A., Gallelli L. (2011 Jul). Pain management for dentists: the role of ibuprofen. Ann Stomatol.

[7] Shamszadeh S., Shirvani A., Eghbal M.J., Asgary S. (2018 Jul). Efficacy of corticosteroids on postoperative endodontic pain: a systematic review and meta-analysis. J Endod.

[8] Mehrvarzfar P, Esnashari E, Salmanzadeh R, Fazlyab M, Fazlyab M. Effect of dexamethasone intraligamentary injection on post-endodontic pain in patients with symptomatic irreversible pulpitis: a randomized controlled clinical trial. Iran Endod J. 2016 Fall;11(4):261-266. https://doi:10.22037/iej.2016.2. 10.22037/iej.2016.2 PMC5069900 27790253

[9] Monteiro L.P.B., Guerreiro M.Y.R., de Castro Valino R., Magno M.B., Maia L.C., da Silva Brandão J.M. (2021 Jan). Effect of intracanal cryotherapy application on postoperative endodontic pain: a systematic review and metaanalysis. Clin Oral Invest.

[10] Vera J., Ochoa-Rivera J., Vazquez-Carcaño M., Romero M., Arias A., Sleiman P. (2015 Nov). Effect of intracanal cryotherapy on reducing root surface temperature. J Endod.

[11] Vera J., Ochoa J., Romero M. (2018 Jan). Intracanal cryotherapy reduces postoperative pain in teeth with symptomatic apical periodontitis: a randomized multicenter clinical trial. J Endod.

[12] Guerreiro M.Y.R., Monteiro L.P.B., de Castro R.F., Magno M.B., Maia L.C., da Silva Brandão J.M. (2021 Mar). Effect of low-level laser therapy on postoperative endodontic pain: an updated systematic review. Compl Ther Med.

[13] Doğanay Yıldız E., Arslan H. (2018 Nov). Effect of low-level laser therapy on postoperative pain in molars with symptomatic apical periodontitis: a randomized placebo-controlled clinical trial. J Endod.

[14] Aminoshariae A., Kulild J.C., Donaldson M., Hersh E.V. (2016 Oct). Evidence-based recommendations for analgesic efficacy to treat pain of endodontic origin: a systematic review of randomized controlled trials. J Am Dent Assoc.

[15] Nagendra babu V., Duncan H.F., Bjørndal L. (2020 Jun). PRIRATE 2020 guidelines for reporting randomized trials in Endodontics: explanation and elaboration. Int Endod J.

[16] Sullivan K.M., Dean A., Soe M.M. (2009). On academics: OpenEpi: a web-based epidemiologic and statistical calculator for public health. Publ Health Rep.

[17] Hariton E., Locascio J.J. (2018 Dec). Randomised controlled trials - the gold standard for effectiveness research: study design: randomised controlled trials. BJOG.

[18] Montero J., Lorenzo B., Barrios R., Albaladejo A., Mirón Canelo J.A., López-Valverde A. (2015 Sep). Patient-centered outcomes of root canal treatment: a cohort follow-up study. J Endod.

[19] O'Keefe E.M. (1976 Oct). Pain in endodontic therapy: preliminary study. J Endod.

[20] Haslerud S., Lopes-Martins R.A., Frigo L. (2017 Jan). Low-level laser therapy and cryotherapy as mono- and adjunctive therapies for achilles tendinopathy in rats. Photomed Laser Surg.

[21] AbuBakr N., Salem Z.A., Ali Z.H., El Assaly M.S. (2018). Comparison of low-level laser versus intra-articular corticosteroid therapy for temporomandibular joint osteoarthritis in rats. Journal of Dentistry Indonesia.

[22] Capps S.G., Brook M. (2009). Cryotherapy and intermittent pneumatic compression for soft tissue trauma. Int J Athl Ther Train.

[23] Malanga G.A., Yan N., Stark J. (2015 Jan). Mechanisms and efficacy of heat and cold therapies for musculoskeletal injury. Postgrad Med.

[24] Al-Nahlawi T., Hatab T.A., Alrazak M.A., Al-Abdullah A. (2016 Dec 1). Effect of intracanal cryotherapy and negative irrigation technique on postendodontic pain. J Contemp Dent Pract.

[25] Gundogdu E.C., Arslan H. (2018 Mar). Effects of various cryotherapy applications on postoperative pain in molar teeth with symptomatic apical periodontitis: a preliminary randomized prospective clinical trial. J Endod.

[26] Silveira Paulo, Silva Luciano, Freitas Tiago, Latini Alexandra (2011). Effects of low-power laser irradiation (LPLI) at different wavelengths and doses on oxidative stress and fibrogenesis parameters in an animal model of wound healing. Laser Med Sci.

[27] AlGhamdi K.M., Kumar A., Moussa N.A. (2012 Jan). Low-level laser therapy: a useful technique for enhancing the proliferation of various cultured cells. Laser Med Sci.

[28] Marshall J.G. (2002). Consideration of steroids for endodontic pain. Endod Top.

[29] Nagendra babu V., Gutmann J.L. (2017). Factors associated with post obturation pain following single-visit nonsurgical root canal treatment: a systematic review. Quintessence Int.

[30] Ng Y.L., Glennon J.P., Setchell D.J., Gulabivala K. (2004 Jun). Prevalence of and factors affecting post-obturation pain in patients undergoing root canal treatment. Int Endod J.

[31] Polycarpou N., Ng Y.L., Canavan D., Moles D.R., Gulabivala K. (2005 Mar). Prevalence of persistent pain after endodontic treatment and factors affecting its occurrence in cases with complete radiographic healing. Int Endod J.

